# Pregnancy-related issues in rare and low-prevalence diseases: results of ERN transversal working group on pregnancy and family planning survey

**DOI:** 10.1186/s13023-024-03435-z

**Published:** 2025-03-10

**Authors:** Dina Zucchi, Diana Marinello, Chiara Tani, Giovanni Fulvio, Silvia Aguilera, Alexandra Benachi, Ruth Biller, Ignacio Blanco, Petra Borgards, Marie-Claude Boiteux, Maria Luisa Brandi, Ester Costafreda, Joao E. Fonseca, Micaela Fredi, Violeta Iotova, Simone Louisse, Cecilia Nalli, Michela Onali, Beverley Power, Christine Rousset-Jablonski, Dominique Sturz, Angela Tincani, Ana Vieira, Susana Capela, Dorica Dan, Julie De Backer, Christine de Die-Smulders, Andreas Dufke, Estelle Lecointe Artzner, Giuseppe Limongelli, Birgit Lorenz, Wiebke Papenthin, María Jesús Pascau, Johanna Raidt, Isabelle Ray-Coquard, Rachel Rimmer, Claas Röhl, Holm Schneider, Tet Yap, Rosaria Talarico, Marta Mosca

**Affiliations:** 1 Rheumatology Unit, Azienda Ospedaliero Universitaria Pisana and Department of Clinical and Experimental Medicine, University of Pisa, ERN ReCONNET, Via Roma 67, Pisa, Italy; 2 Department of Medical Biotechnologies, University of Siena, Siena, Italy; 3 Spanish Association for Antiphospholipid Syndrome (SAF España), ERN ReCONNET, Madrid, Spain; 4 Service de Gynecologie Obstetrique, Hopital Antoine Béclère, AP-HP, Université Paris Saclay, ERN ERNICA, Clamart, France; 5 ERN GUARD-Heart, Coordinator Center, Amsterdam, The Netherlands; 6 ARVC-Selbsthilfe E.V., Munich, Germany; 7 Germans Trias Hospital, ERN GENTURIS, Badalona, Spain; 8 VASCERN, Coordinator Center, Paris, France; 9 Cutis Laxa Internationale, ERN-Skin, Bons-en-Chablais, France; 10 Università Vita-Salute San Raffaele, ERN BOND, Milan, Italy; 11 SAMS Association, ERN GUARD-Heart, Philadelphia, USA; 12 Serviço de Reumatologia Centro Hospitalar Universitário Lisboa Norte, ERN ReCONNET, Lisbon, Portugal; 13 Instituto de Medicina Molecular, Faculdade de Medicina, Universidade de Lisboa, Centro Académico de Medicina de Lisboa, Lisbon, Portugal; 14 Department of Clinical and Experimental Sciences, Rheumatology and Clinical Immunology, University of Brescia, ASST Spedali Civili of Brescia, ERN ReCONNET, Brescia, Italy; 15 Department of Pediatrics, UMHAT “St. Marina”, Endo-ERN, Varna, Bulgaria; 16 ERN Euro-NMD, Coordinator Center, Paris, France; 17 CDH UK-The Congenital Diaphragmatic Hernia Charity, ERNICA, King’s Lynn, UK; 18 Department of Surgery, Leon Berard Cancer Center, Lyon, France; 19 INSERM U1290 RESHAPE (RESearch in HealthcAre PErformance), EURACAN, Lyon, France; 20 ERN-Eye, Coordinator Center, Strasbourg, France; 21 Liga Portuguesa Contra as Doenças Reumáticas, Núcleo de Sjögren, ERN ReCONNET, Lisbon, Portugal; 22 ERN ITHACA, Coordinator Center, Paris, France; 23 Department of Cardiology and Center for Medical Genetics, Ghent University Hospital Belgium, VASCERN, Ghent, Belgium; 24 GROW, School for Oncology and Reproduction, Maastricht University, ERN NMD, Maastricht, Netherlands; 25 Institute of Medical Genetics and Applied Genomics, University of Tübingen, Tübingen, Germany; 26 Centre for Rare Diseases, University of Tübingen, ERN ITHACA, Tübingen, Germany; 27 Sarcoma Patients Advocacy Global Network (SPAGN), ERN EURACAN, Berlin, Germany; 28 Inherited and Heart Disease Unit, Monaldi Hospital, AO Colli-University of Campania “Luigi Vanvitelli”, ERN GUARD-Heart, Naples, Italy; 29 ERN RARE-LIVER, Wilson Working-Group, Pittsburgh, USA; 30 Paediatric Transplant Unit, La Paz University Hospital, ERN Transplantchild, Madrid, Spain; 31 Department of General Pediatrics, University Hospital Muenster, ERN LUNG, Muenster, Germany; 32 Centre Leon Bérard, Université Claude Bernard Lyon Est, ERN EURACAN, Lyon, France; 33 ERN RITA, Coordinator Center, Utrecht, The Netherlands; 34 NF Kinder, Vienna, Austria; 35 University Hospital Erlangen, ERN Skin, Erlangen, Germany; 36 Guy’s and St Thomas’ NHS Trust, ERN eUROGEN, London, UK

**Keywords:** Rare diseases, Pregnancy-related issues, Unmet needs

## Abstract

**Background:**

Rare and complex diseases can have a significant impact on family life, and managing the reproductive aspects of patients of childbearing age with rare diseases is often difficult and complex.

A European Reference Network (ERN) Transversal Working Group (WG) on Pregnancy and Family Planning was created to join forces to promote and address issues on these topics in rare and low-prevalence diseases.

**Objective:**

To outline the challenges and the good practices related to pregnancy and family planning in rare and complex diseases for healthcare professionals (HCPs).

**Methods:**

A survey on state of the art and unmet needs was created by a co-design group of both clinicians and patients’ representatives from 20 ERNs. The survey was uploaded in English on the online platform “EU Survey” and disseminated by respective ERNs and learned societies. Seven transversal domains were explored in the survey by using closed and open-ended questions: fertility preservation, pre-conceptional counselling, family planning counselling, pre-implantation diagnosis, prenatal diagnosis, pregnancy monitoring and post pregnancy monitoring, lactation monitoring/counselling and newborn management. The questions investigated for each topic were the following: level of importance, activities performed by the centre, clinical challenges, good practice and educational activities.

**Results:**

A total of 197 answers were collected from 24 different countries. Unmet needs for HCPs included: the need to improve communication between different HCPs, the lack of predefined organizational pathways, the lack of availability of expert HCPs for some pregnancy-related issues and the need to streamline the care provided among different countries. In addition, the survey underlined the need to improve the educational activities provided to rare disease patients.

**Conclusions:**

Physicians and patients need to be educated on the emerged unmet needs in order to standardize the information for both HCPs and patients with rare diseases. Educational activities should be considered to help to disseminate information.

**Supplementary Information:**

The online version contains supplementary material available at 10.1186/s13023-024-03435-z.

## Background

Family planning, pregnancy, childbirth, postnatal period and the management of a newborn child represent complex moments in the life of every woman and family. Managing such a delicate time can be extremely more complex if one lives with a rare disease that can have a significant impact on each of these aspects of family life [1–3].

From the physician's point of view, dealing with and managing the reproductive aspects of patients of childbearing age with rare diseases is often difficult and complex.

One of the main reasons is that there is often a lack of standardised guidelines and protocols for addressing these aspects in patients with rare diseases.

European Reference Networks (ERNs) were launched by the European Commission in 2017 with the aim of tackling low prevalence and rare diseases that require highly specialised treatment and promoting concentration of knowledge and resources through virtual networks involving healthcare providers (HCPs) across the European Union (EU) [4, 5].

An ERN Transversal Working Group (WG) on Pregnancy and Family Planning was created to join forces to promote and address issues on pregnancy and family planning in rare and low-prevalence diseases. The initial actions of the WG included the creation of a survey on state of the art and unmet needs in family planning, reproduction and related themes in order to plan future transversal activities based on the results of the survey.

The aim of this work was to describe the survey results for HCPs to outline the challenges and the good practices related to pregnancy and family planning in rare and complex diseases.

## Methods

A co-design group of both clinicians and patients’ representatives from different ERNs was created and members from different ERNs were engaged, ensuring the transversality of the surveys.

In the WG 20 ERNs were represented: ERN on bone disorder (ERN BOND), ERN on craniofacial anomalies and ear, nose and throat disorders (ERN CRANIO), ERN on endocrine conditions (Endo-ERN), ERN on epilepsies (EpiCARE), ERN on inherited and congenital anomalies (ERNICA), ERN on respiratory diseases (ERN LUNG), ERN on skin disorders (ERN Skin), ERN on adult cancers (EURACAN), ERN on urogenital diseases and conditions (ERN Eurogen), ERN on neuromuscular diseases(ERN EURO-NMD), ERN on eye diseases (ERN EYE), ERN on genetic tumour risk syndromes (ERN GENTURIS), ERN on diseases of the heart (ERN GUARD-Heart), ERN on congenital malformations and rare intellectual disability (ERN ITHACA), ERN on paediatric cancer (ERN PaedCan), ERN on hepatological diseases (ERN RARE-LIVER), ERN on connective tissue and musculoskeletal diseases (ERN ReCONNET), ERN on immunodeficiency, autoinflammatory and autoimmune diseases (ERN RITA), ERN on Transplantation in Children (ERN TRANSPLANT-CHILD) and ERN on Rare Multisystemic Vascular Diseases (VASCERN). Each ERN nominated 1 clinician and 1 European Patient Advocacy Groups (ePAG) Advocate to represent the Network and bring back the discussion to the ERN.

The co-design group discussed the preliminary drafts of the survey, and an updated version was then provided following the comments provided by the co-design group.

The group identified 7 transversal domains to be explored in the surveys:

- Fertility preservation
- Pre-conceptional counselling
- Family Planning counselling
- Pre-implantation diagnosis
- Prenatal diagnosis
- Pregnancy monitoring
- Post pregnancy monitoring, lactation monitoring/counselling, newborn management

The survey included closed and open-ended questions and mainly explored for each domain: level of importance (response options were: very important, important, moderately important, slightly important and not important), activities performed by the centre, clinical challenges, good practice and educational activities related to the selected topics.

The complete questionnaire, specifically designed for HCPs, is listed in supplementary material S1. For each domain there were a maximum of 9 questions.

The survey was anonymous and was developed in English on the online platform “EU Survey” [6]. HCPs survey was mainly disseminated by respective ERNs and scientific and professional societies, while patient’s survey was made available across social media as well as across different patients’ associations, also with the support of the panel of patients’ representatives.

The survey was launched on 28th February and closed on 31 July 2022.

Since the survey was completely anonymous and no personal information was collected, an approval of the Institutional Board Review was not needed and the participant’s consent to the study was obtained by replying to the survey.

The answers were evaluated using descriptive statistics, and the results of a preliminary analysis were shared and discussed with the members of WG after closing the survey.

## Results

After the launch of the survey, a total of 197 answers from HCPs were collected with different contributions from the ERNs (Table 1).

**Table 1:** ERN of the respondents

Name of the ERN selected by the respondents	N	%
VASCERN	36	14.94%
ENDO-ERN	33	13.69%
ERN ReCONNET	23	9.54%
EURACAN	23	9.54%
EURO-NMD	22	9.13%
ERN GENTURIS	16	6.64%
ERN PaedCan	14	5.81%
RARE-LIVER	14	5.81%
ERN-EuroBloodNet	11	4.56%
ERN BOND	8	3.32%
GUARD-HEART	7	2.91%
ERNICA	6	2.49%
ERN ITHACA	5	2.07%
ERN RND	5	2.07%
ERN CRANIO	4	1.66%
MetabERN	4	1,66%
TransplantChild	4	1.66%
ERN ERKNET	2	0.83%
ERN RITA	2	0.83%
EUROGEN	1	0.42%
ERN LUNG	1	0.42%

In some cases more than one ERN was selected in the same answer

Respondents were from 24 different countries (Austria, Belgium, Bulgaria, Czechia, Croatia, Cyprus, Denmark, Finland, France, Germany, Greece, Hungary, Ireland, Italy, Latvia, Lithuania, Luxemburg, Malta, Netherlands, Poland, Portugal, Slovenia, Spain, Sweden), and the majority were from Italy (21.3%). In addition, 8 answered “Other” at question “In which country is your centre based?”.

The level of importance provided to each domain by the respondents are detailed in Table 2, and the percentage of the answers “very important/important” and “not important” on the total responses for each ERN are reported in Supplementary Material (Figure S2–S6).

**Table 2:** Rate of relevance of the explored topics

	Rate of relevance (% of respondents)		
Topic	Very important/important	Moderately important/slightly important	Not important
Fertility preservation	121 (61.42%)	51 (25.89%)	25 (12.69%)
Pre-conceptional counselling	164 (83.25%)	25 (12.68%)	8 (4.06%)
Family planning counselling	141 (71.57%)	45 (22.85%)	11 (5.58%)
Pre-implantation diagnosis	100 (50.76%)	58 (29.44%)	39 (19.80%)
Prenatal diagnosis	106 (53.81%)	62 (31.47%)	29 (14.72%)
Pregnancy monitoring	149 (75.63%)	30 (15.22%)	18 (9.14%)
Post pregnancy monitoring	132 (67.01%)	43 (21.83%)	22 (11.17%)
Lactation monitoring/counselling	94 (47.72%)	62 (31.47%)	41 (20.81%)
New-born management	120 (60.91%)	47 (23.86%)	30 (15.23%)

Overall, the topics indicated as important by the highest percentage of respondents were “pre-conceptional counselling” and “pregnancy monitoring” (83.3% and 75.6%, respectively), while the lower percentage of “very important/important” was recorded for lactation monitoring/counselling and pre-implantation diagnosis (47.7% and 50.8%, respectively).

### Fertility preservation, pre-conceptional counselling and family planning counselling

Fertility preservation was considered very important/important from 61.2% of the respondents, and not important for 12.7%.

The fragmentation of care in several centers was pointed out, and the challenges which emerged from the open questions were timing (especially in cases in which it was necessary to start therapy quickly) and costs (not always covered by National Health Systems).

Pre-conceptional counselling, defined as a counselling before a planned pregnancy, was the topic with the highest rate of importance, since 83.3% of the respondents indicated it as “very important/important”, with 100% as outcome on the same question for 8 ERNs (Fig. 2 in Supplementary Material). For only 4.1% of participants the topic was rated as not important.

Organizational issues were the most highlighted challenges for pre-conceptional counselling, and in particular the fact that specialists such as gynecologists and/or geneticists are not always in the same hospital.

Family planning counselling, defined as discussion of contraception or planning the time of pregnancy attempt also related to disease activity, was rated as very important/important by 71.6%, and not important by 5.6% of participants. In the same topic, the answers to open-ended questions underlined the difficulties in communication between different HCPs, when multidisciplinary teams were not available, and the organizational issues, in particular the lack of standardized protocols.

### Pre-implantation diagnosis, prenatal diagnosis and pregnancy monitoring

Approximately half of the responses (50.8%) rated pre-implantation diagnosis as very important/important, while 14.7% rated this topic as not important.

The most common problems described in the open-ended questions were that not all centres/countries perform these procedures and that costs can be high. In addition, regulations may differ from one country to another, and the indications authorised by ethics committees are not the same in all countries.

Similar rates of importance were recorded for prenatal diagnosis (53.8% very important/important, 19.8% not important). Among the services provided by the centers regarding this domain there were screening for fetal aneuploidy, echocardiographic monitoring for patients SSA/SSB + and genetic counselling. However, in several centers procedures are scarcely available.

Pregnancy monitoring was rated as very important/important by about 75%, and not important by less than 10% of participants. Not all the centers have multidisciplinary teams, and organizational issues and lack of expertise in some hospitals was described in open-ended questions.

### Post-pregnancy monitoring, lactation monitoring/counselling, newborn management and educational activities

Topics regarding the period after the delivery, the puerperium, were important for 47.7% to 67.0% of participants, with the lower percentage for lactation monitoring/counselling and the highest for post-pregnancy monitoring.

Lack of standardized protocols caused the most common problem related to these topics.

From the survey it emerged that less than 50% of centers provide topic-related educational activities to patients with rare diseases.

Educational activities were provided from a minimum of 29.4% to a maximum of 46.2% of cases on account of the different topics, with the lower percentage for pre-impantation diagnosis and the higher for pre-conceptional counselling. The most frequent kind of educational activities were leaflets/brochures and/or specific face to face educational sessions. In Table 3 there are detailed for each kind of educational activities the minimum and maximum percentage calculated on the total number of answers and regardless of the ERNs.

**Table 3:** Educational activities

Kind of educational activities provided to patients	% (min to max)
Leaflets/brochure and educational materials	5.92–12.27%
Specific face to face education sessions	16.18–20.30%
On-line educational material	2.55–5.39%
Link to useful website	2.59–6.32%
Contact to patient organisation(s) or patient forum	3.02–8.92%
Other	1.24–6.17%
NA	39.78–65.95%

## Discussion

Despite the progress in approach to rare diseases, there are still many unmet needs for rare patients, families and caregivers [7], including the management of pregnancy and family planning.

The aim of this work was to analyse the results of an ERN-wide surveys for HCPs launched among different ERNs to promote and address issues on pregnancy and family planning in rare and low-prevalence diseases**.**

**Fig. 1:**
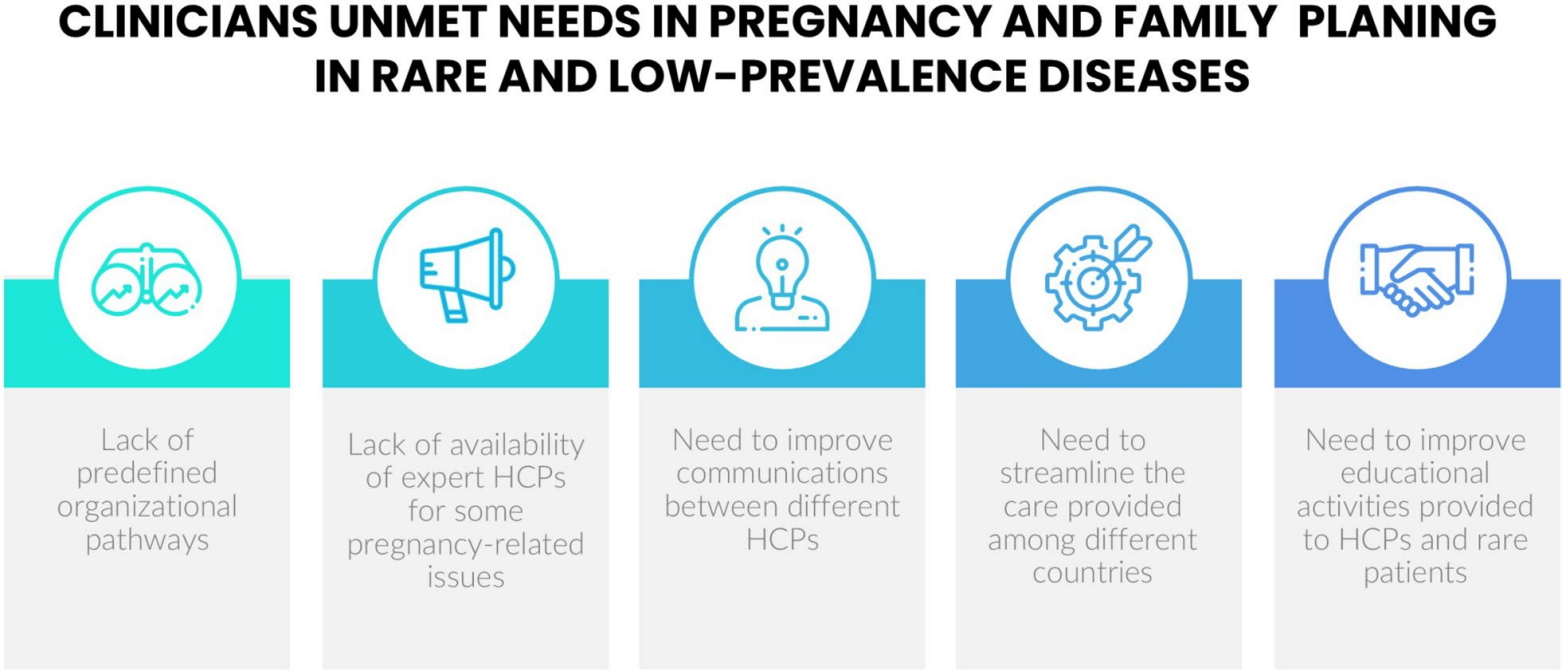
clinicians unmet needs in pregnancy and family planning in rare and low-prevalence diseases. *HCPs* healthcare professionals

Overall, the results of the survey pointed out some unmet needs for HCPs, and in particular: the need to improve communications between different HCPs and educational activities provided to rare patients, the lack of predefined organizational pathways and of availability of expert HCPs for some issues and the need to streamline the care provided among different countries. Main unmet needs emerged from the survey are reported in Fig. 1.

The importance of multidisciplinary teams and of collaboration between different HCPs with expertise in pregnancy in rare diseases emerged as a very important aspect for all the explored domains. This is in line with previous results related also to other more common medical conditions, for which multidisciplinary team management is recommended to reduce the occurrence of adverse maternal and fetal outcomes [8]. This aspect is clearly demonstrated by a recent systematic review on patients with Systemic Lupus Erythematosus showing that a multidisciplinary care during pregnancy had a positive impact on pregnancy-related complications, on the disease course, and relieved the patients’ psychological impact [9].

This is related to the necessity to give the right information/support to the patients and to improve the awareness on pregnancy-related issues. Starting the journey with an appropriate pre-conceptional counselling could be the best way to achieve this aim, but identify the right timing for this kind of counselling is still challenging.

The results related to almost all domains underlined that there is no standardization in the approach to rare patients, and lack of standardized protocols emerged especially for the topics regarding the period after the delivery. The lack of guidelines could be a possible explanation, and developing organizational pathways may help to homogenize the care provided to rare patients in different clinical settings. With regards to rare and complex rheumatic diseases, the absence of formalized care pathways related to pregnancy and family planning emerged also in a previous work aimed at analyzing the answers of HCPs from European and non-European referral centers [10].

For several domains, and especially for fertility preservation, pre-implantation diagnosis and prenatal diagnosis, common issues are the scarce knowledge on these techniques, the lack of centers/countries which provide them and the high costs, not always covered by the National Health Service. In addition, the presence of religious, cultural, and ethical issues remains a challenge.

To improve knowledge on these topics and to standardize the approach to rare and low-prevalence patients, educational activities should be considered to help to disseminate general information. For instance, developing links to online resources such as leaflets in electronic version, or providing contacts for patient groups will certainly help in addressing many issues that emerged from the survey.

For families dealing with prenatal rare diseases, patient advocacy organizations was reported to be very useful for parents also to help in alleviating some of their stress [11], and in cases of children with rare diseases the importance of specific support for all family members and of HCPs training was previously reported (3). Overall, our study showed transversal needs that can be addressed through a standardized approach from an organizational perspective, and that will then need to be personalized according to disease-specific needs.

We acknowledge that in the survey there was no standardization or guidelines for the HCPs when asked to rate the relevance of a topic in their area; what we are proposing is based on expert opinion and this aspect may represent a weakness in the work. However, we think that HCPs expert opinion can provide a useful basis and a necessary starting point to plan future research and initiatives for rare and low-prevalence diseases.

## Conclusions

Overall, the results pointed out the need to educate both physicians and patients on the basis of the emerging unmet needs. Online resources can be an excellent educational tool, they may help in disseminating and standardizing educational activities in order to homogenize the information for HCPs and patients. Therefore, initiatives in this direction by scientific societies, ERNs and patient associations should be promoted and encouraged.

We acknowledge that there is heterogeneity in patients, diseases, settings and countries but, to our knowledge, this is the first work that pointed out challenges related to pregnancy and family planning regarding all these rare and complex diseases. Overall, all the information matters to improve the management of these diseases.

## Supplementary Information

Additional file1.

Additional file2.

Additional file3.

Additional file4.

Additional file5.

Additional file6.

## Data Availability

The dataset generated and analysed during the current study is not publicly available to keep the answers to open questions confidential but is available from the corresponding author on reasonable request.

## Abbreviations

ERNs: European reference networks
HCPs: Healthcare providers
EU: European Union
WG: Working group
ERN BOND: European Reference Network on bone disorder
ERN CRANIO: European Reference Network on craniofacial anomalies and ear, nose and throat disorders
Endo-ERN: European Reference Network on endocrine conditions
EpiCARE: European Reference Network on epilepsies
ERNICA: European Reference Network on inherited and congenital anomalies
ERN LUNG: European Reference Network on respiratory diseases
ERN Skin: European Reference Network on skin disorders
EURACAN: European Reference Network on adult cancers
ERN Eurogen: European Reference Network on urogenital diseases and conditions
ERN EURO-NMD: European Reference Network on neuromuscular diseases
ERN EYE: European Reference Network on eye diseases
ERN GENTURIS: European Reference Network on genetic tumour risk syndromes
ERN GUARD-Heart: European Reference Network on diseases of the heart
ERN ITHACA: European Reference Network on congenital malformations and rare intellectual disability
ERN PaedCan: European Reference Network on paediatric cancer
ERN RARE-LIVER: European Reference Network on hepatological diseases
ERN ReCONNET: European Reference Network on connective tissue and musculoskeletal diseases
ERN RITA: European Reference Network on immunodeficiency, autoinflammatory and autoimmune diseases
RN TRANSPLANT-CHILD: European Reference Network on Transplantation in Children
VASCERN: European Reference Network on Rare Multisystemic Vascular Diseases
