# Supplementary figures and images for: Pregnancy-related issues in rare and low-prevalence diseases: results of ERN transversal working group on pregnancy and family planning survey
Source: Orphanet J Rare Dis. 2025 Mar 10;20:112. doi: 10.1186/s13023-024-03435-z (PMC11892229; doi:10.1186/s13023-024-03435-z)

**Supplementary Material S1**: complete questionnaire


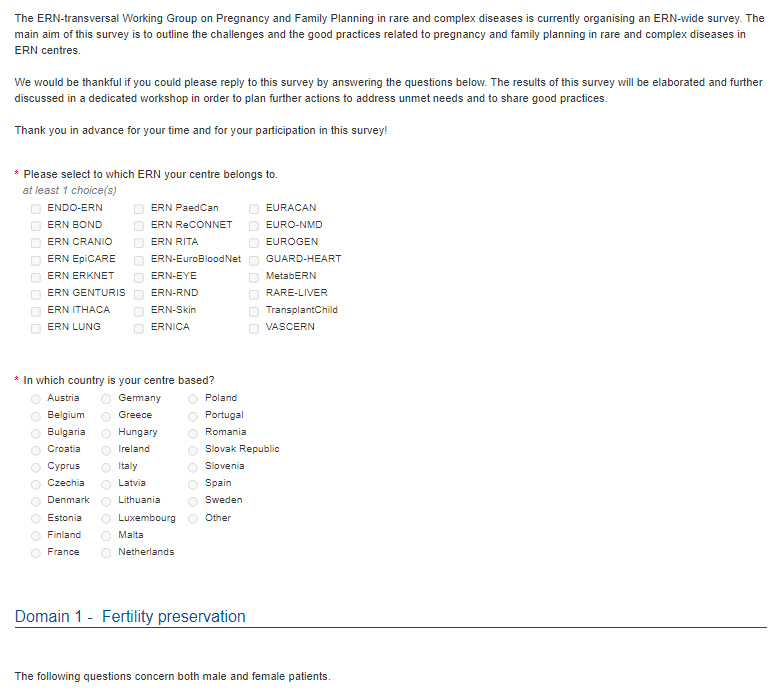

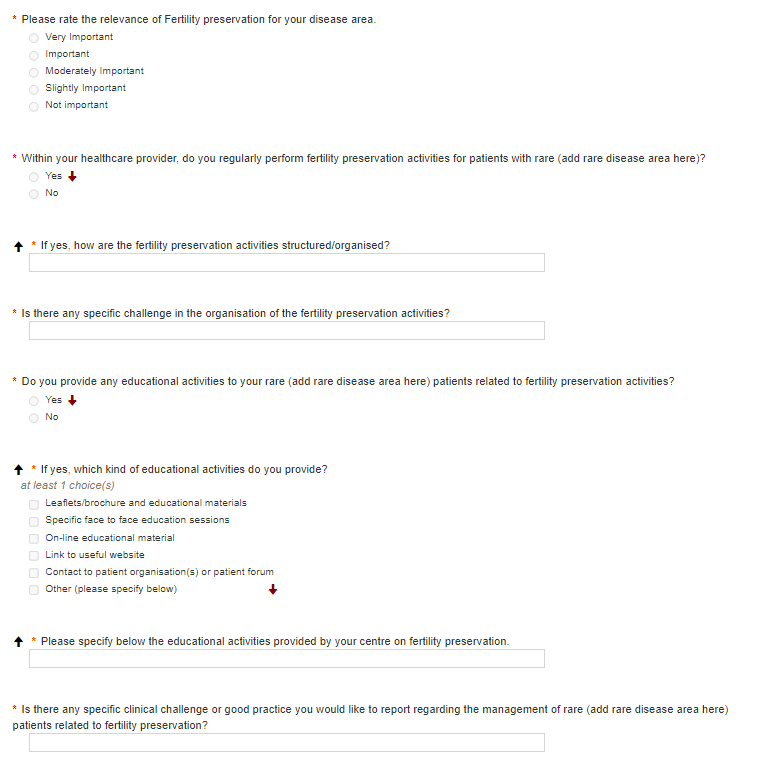


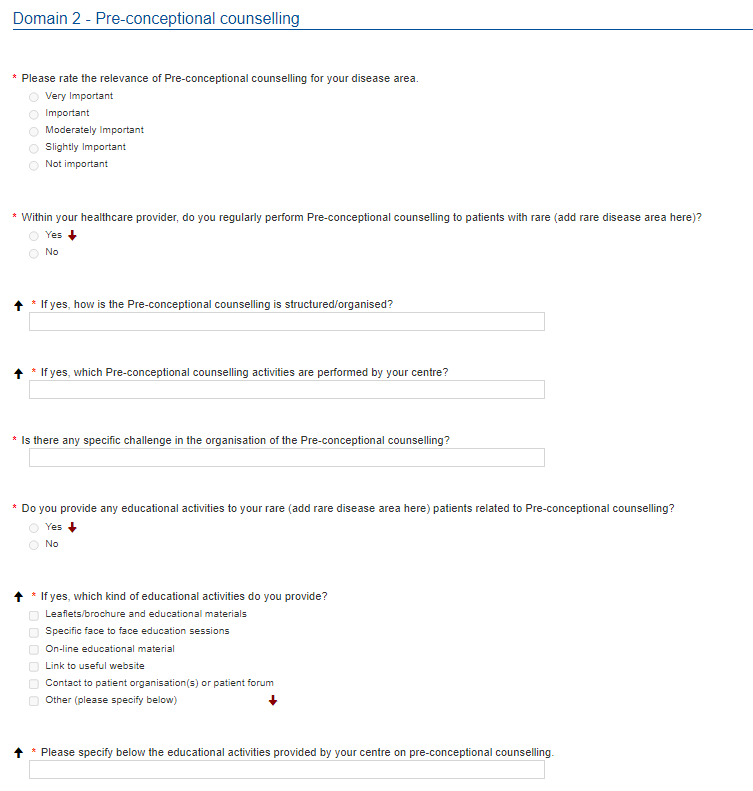

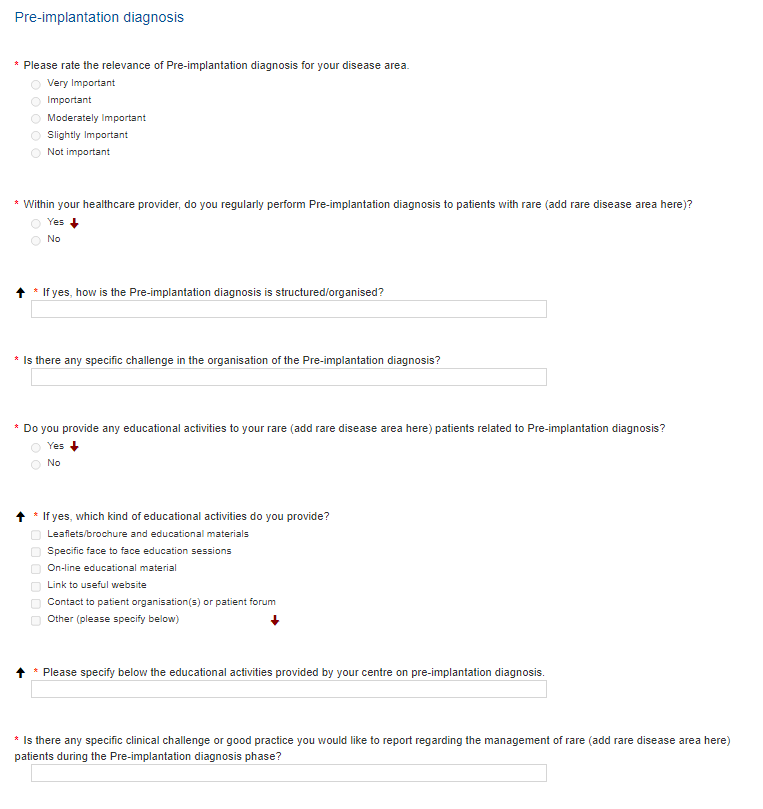


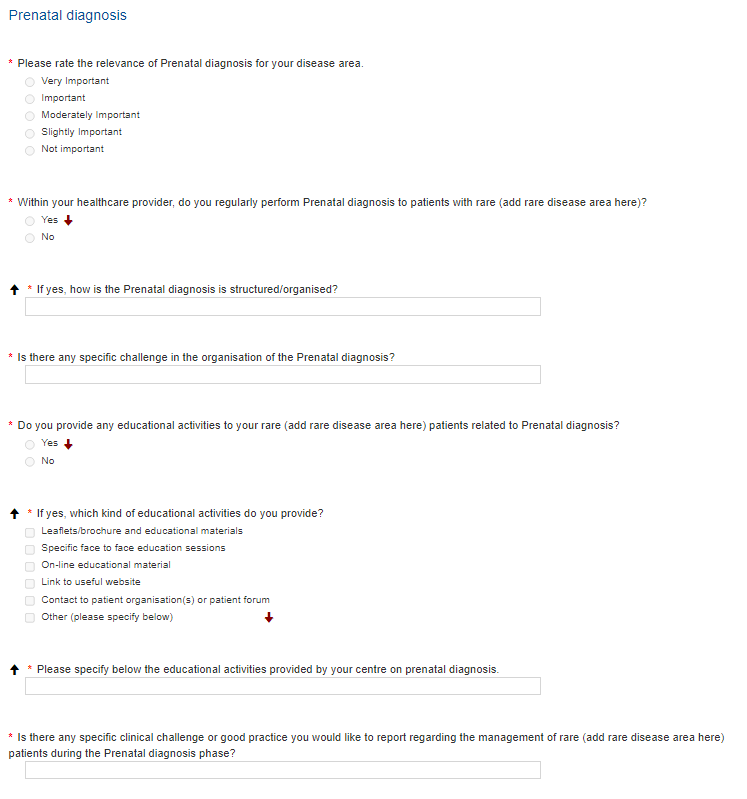


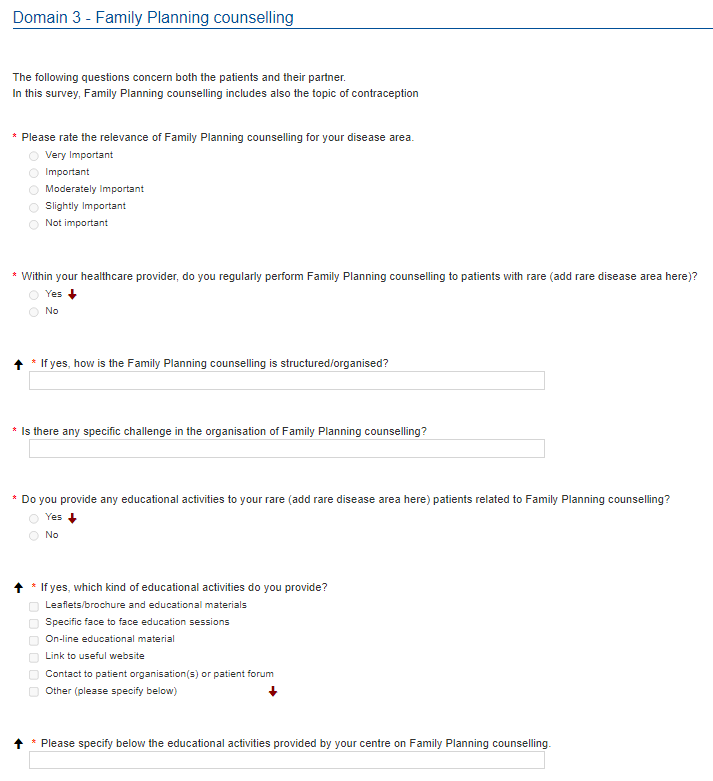

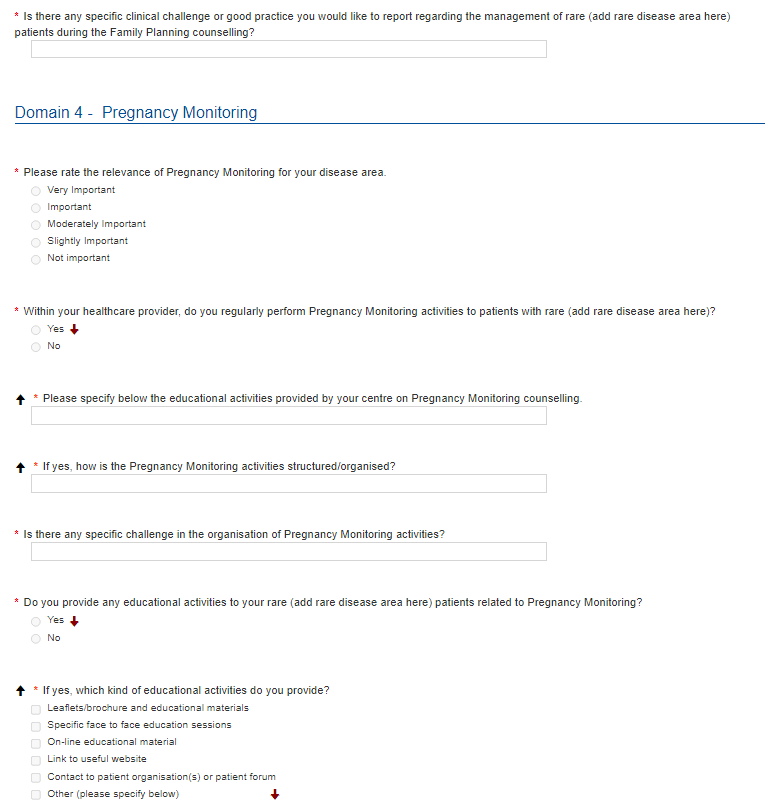

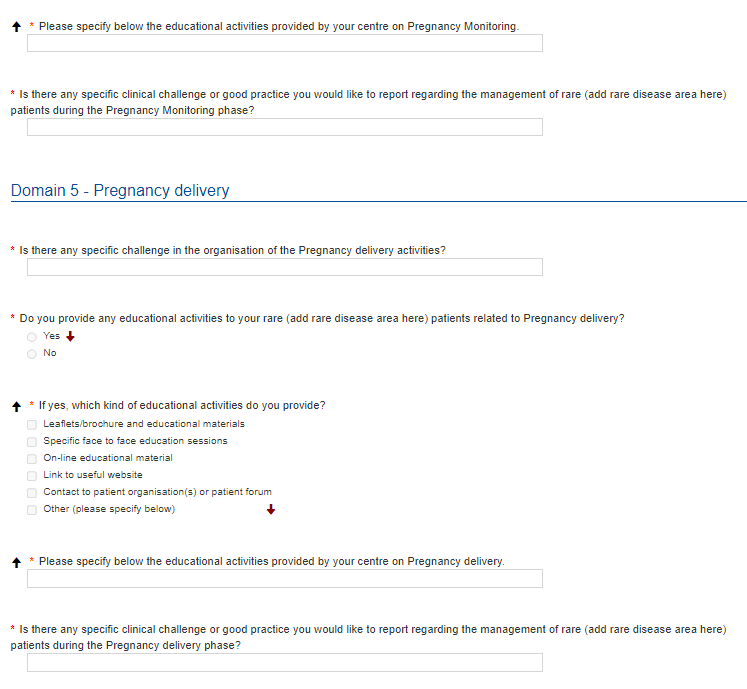

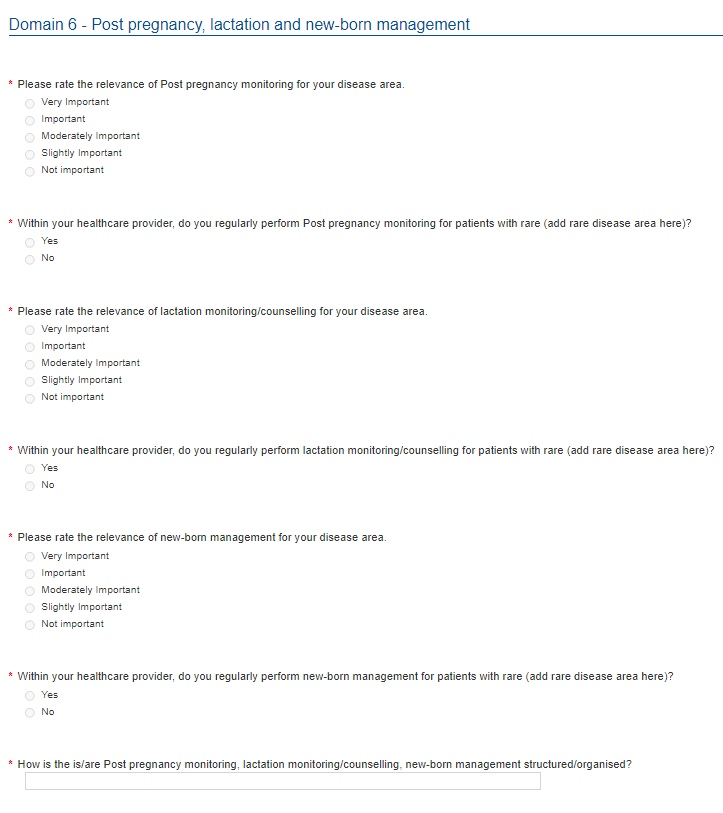

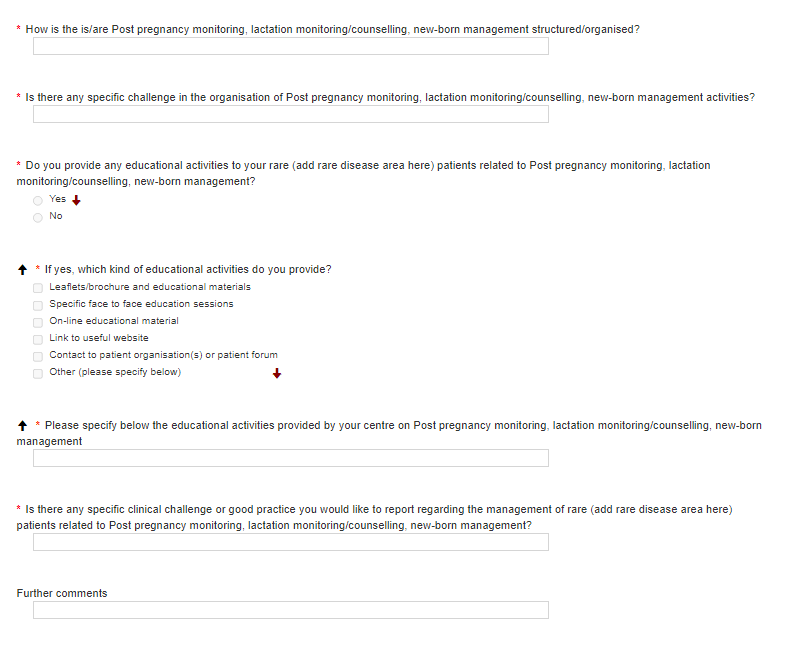

Supplement: Supplementary file 1 — Additional file1. [file 13023_2024_3435_MOESM1_ESM.docx]
