# Supplementary material for: Pregnancy-related issues in rare and low-prevalence diseases: results of ERN transversal working group on pregnancy and family planning survey
Source: Orphanet J Rare Dis. 2025 Mar 10;20:112. doi: 10.1186/s13023-024-03435-z (PMC11892229; doi:10.1186/s13023-024-03435-z)

**Figure S5:** percentage of the answers “very important/important” and “not important” on the total responses for each ERN for “Post pregnancy monitoring” and “Lactation monitoring/counselling”


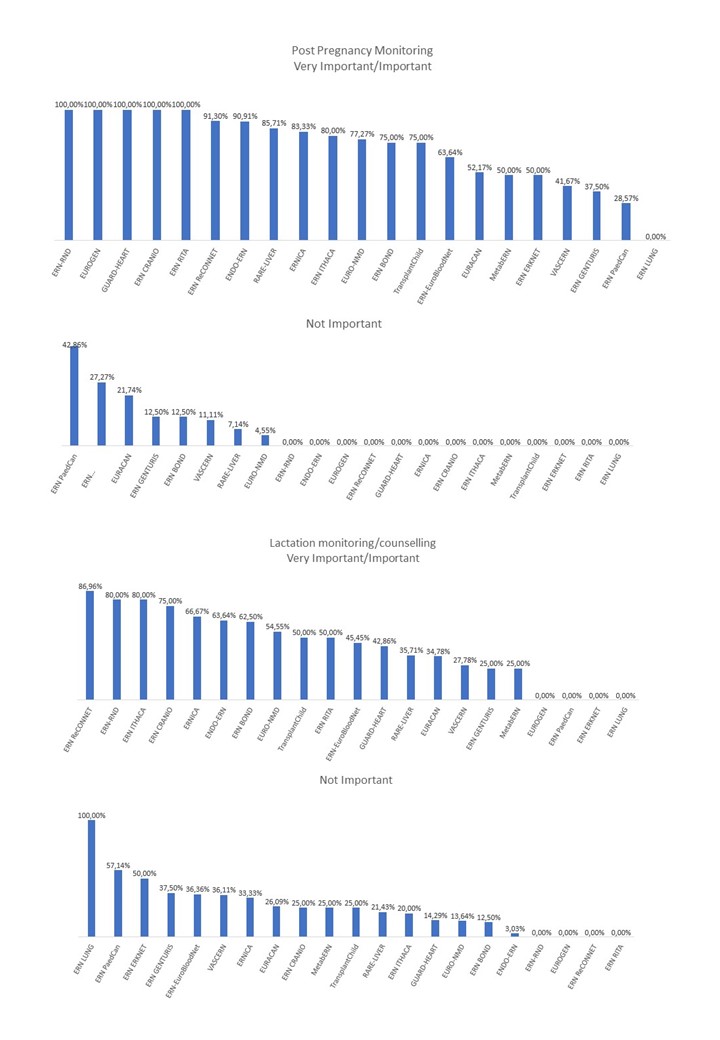

Supplement: Supplementary file 5 — Additional file5. [file 13023_2024_3435_MOESM5_ESM.docx]
